# Evaluating preoperative risk factors for deep vein thrombosis in elderly patients with hip fractures and chronic kidney disease: a retrospective study

**DOI:** 10.1186/s40001-025-03409-2

**Published:** 2025-11-21

**Authors:** Ruili Jia, Xiaoqian Men, Fang Ran, Xiaodong Li, Yubin Long

**Affiliations:** 1 Department of Nephrology, Baoding First Central Hospital of Hebei Medical University, Baoding, China; 2 Department of Ultrasound, The Third Hospital of Hebei Medical University, Shijiazhuang, China; 3 Department of Orthopedics, Baoding No.1 Central Hospital, Baoding, Hebei Province China

**Keywords:** Chronic kidney disease, Hip fractures, Deep venous thrombosis, Age, D-dimer, White blood cells

## Abstract

**Background:**

Deep vein thrombosis (DVT) represents a significant and potentially lethal complication in bedridden elderly inpatients, leading to severe disability and mortality.

**Purpose:**

This study aimed to identify determinants contributing to DVT incidence in elderly patients with concurrent hip fractures and chronic kidney disease (CKD).

**Methods:**

We collected comprehensive data from elderly patients diagnosed with hip fractures and CKD at our hospital between November 2015 and January 2023. Patients were categorized into DVT and non-DVT groups. We conducted detailed demographic, comorbidity, and laboratory analyses using univariate and logistic regression methods. Receiver-operating characteristic (ROC) curves were utilized to establish critical thresholds for predictive variables.

**Results:**

The study revealed a 34.4% DVT rate among 180 patients. Univariate analysis identified significant associations between DVT and older age (*p* = 0.031), elevated white blood cell (WBC) count (*p* = 0.005), prolonged thrombin time (TT) (*p* = 0.030), and increased D-dimer levels (*p* < 0.001). Logistic regression showed that age (*p* = 0.009, odds ratio [OR] = 1.049), WBC count (*p* = 0.001, OR = 1.131), and D-dimer levels (*p* = 0.010, OR = 1.190) were independent predictors of DVT. ROC analysis established critical thresholds: age (73 years), D-dimer (3.3 ng/mL), and WBC count (9.5 × 10^9/μL).

**Conclusions:**

Age, D-dimer, and WBC levels independently predict DVT in elderly patients with hip fractures and CKD. Precise thresholds facilitate personalized DVT risk assessment, enabling early and targeted interventions.

## Introduction

By 2050, the global population is projected to age significantly, with the number of individuals aged 60 and over expected to reach 2 billion [1]. This demographic trend not only signifies an increase in chronic health issues but also markedly raises the incidence of serious health conditions in the elderly, particularly hip fractures and chronic kidney disease (CKD). Hip fractures pose a significant risk to the health and quality of life of the elderly. Besides, based on the Centers for Disease Control and Prevention (CDC)’s data, CKD prevalence significantly rises with age, being 34% in those 65 and older, much higher than the 12% in the 45–64 group and 6% in 18–44 years old. This highlights the increased CKD risk in the elderly. As kidney function deteriorates, there is a corresponding rise in fracture rates among older individuals who experience falls [2]. Renal osteodystrophy (ROD), a critical public health issue in aging populations, significantly complicates the management of hip fractures in patients with chronic kidney disease. This condition not only exacerbates the severity of fractures but also poses challenges in post-fracture recovery, thereby profoundly affecting overall patient health [3]. In elderly patients with hip fractures and chronic kidney disease, severe pain and reduced mobility are common, often resulting in extended recovery times. Studies show that factors like inflammation and blood clotting play a role in the link between lower kidney function and higher VTE risk. Exercise, statins, and warfarin can lower VTE risk for people with or without CKD, but a normal BMI does not reduce VTE risk in CKD patients, highlighting the complex relationship between CKD and VTE risk factors [4]. This combination of conditions, particularly decreased mobility and the systemic impact of kidney disease, not only affects overall health but also significantly increases the risk of developing deep vein thrombosis (DVT) as a further complication.

DVT, often arising as a serious complication in lower extremity fractures, can lead to disability and life-threatening pulmonary embolism [5]. In the absence of early intervention or effective management, DVT can progress to cause enduring complications, such as chronic pain, subsequent varicose veins, and ulcers, with a serious risk of developing pulmonary embolism. Moreover, long-term effects like post-thrombotic syndrome and chronic thromboembolic pulmonary hypertension from DVT are significant contributors to disability. The incidence of DVT in elderly patients with hip fractures reaches as high as 17.1%, and the 30-day mortality rate stands at 5.1%. This underscores the critical nature of vigilant monitoring and management for DVT in this vulnerable population to mitigate associated risk [6]. Zhang et al.’s study indicates a 32.8% incidence of DVT in elderly patients with simple hip fractures, highlighting its significant prevalence [7]. Research [8] identifies key risk factors, such as age, delayed hospital admission, post-injury surgery, and so on. Besides, DVT diagnosis often involves coagulation markers like D-dimer, fibrinogen, and platelet count, with high D-dimer levels suggesting a risk of DVT [9]. However, the applicability and effectiveness of D-dimer levels as a diagnostic tool in elderly patients with fractures are still subject to ongoing debate and controversy [10].

It is widely recognized that early diagnosis of DVT is vital, as identifying predictors post-hospital admission can significantly aid in its prevention. Despite the recognized importance of DVT research, there remains a notable gap in studies specifically targeting DVT in elderly patients with concurrent hip fractures and CKD, an area our research aims to address. Our study seeks to identify risk factors linked to DVT prevalence in this demographic and investigate the potential impact of D-dimer levels on DVT incidence, ultimately enhancing our understanding and informing improved prevention strategies. The findings of this study have the potential to inform clinical practices and public health policies, ultimately improving the management and prevention of DVT in a population vulnerable due to age and comorbid conditions.

## Materials and methods

### Ethics statement

For this research investigation, we conducted a retrospective analysis of electronic medical records encompassing elderly patients who were diagnosed with both hip fractures and CKD and who received treatment at our hospital from November 2015 to January 2023. Our study was conducted in accordance with ethical standards outlined in the 1964 Declaration of Helsinki, and it received approval from the institutional review board of Baoding First Central Hospital (2022064). Due to the retrospective nature of this study, formal informed consent from the patients was not deemed necessary.

### Patients

**Fig. 1:**
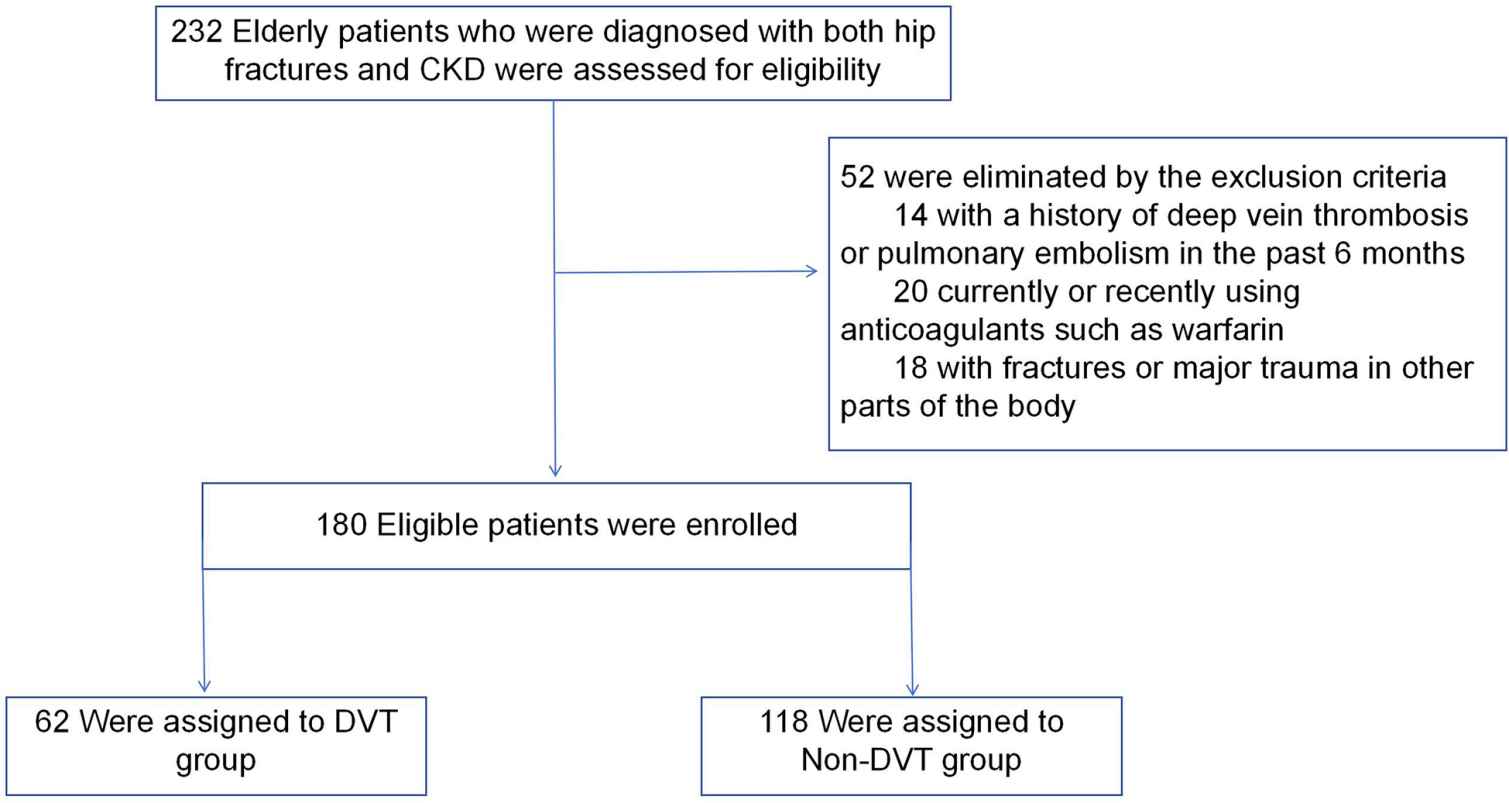
Exclusion criteria and the eligible cases included in this study

This retrospective study was carried out in our Hospital, a tertiary hospital with a level I trauma center. Data from 180 patient’s electronic medical records were extracted following the inclusion and exclusion criteria. Inclusion criteria were as follows: (1) age 60 years or older, (2) confirmed diagnosis of CKD, (3) definite diagnosis of hip fracture supported by imaging, and (4) availability of complete and comprehensive patient data. Meanwhile, exclusion criteria were implemented to ensure the specificity of our study group: (1) exclusion of patients with a history of DVT or pulmonary embolism (PE) in the past 6 months, (2) exclusion of currently or recently using anticoagulants such as warfarin, and (3) exclusion of individuals with fractures or major trauma in other parts of the body. These criteria were meticulously designed to refine our study cohort and allow us to investigate the relationship between hip fractures and CKD in a targeted population of elderly individuals. (Fig. 1).

CKD was identified based on a documented physician diagnosis in the electronic medical record. CKD stage (KDIGO G categories) was not available in our dataset and was therefore not included in the analyses. Per institutional practice, pharmacologic DVT prophylaxis was generally indicated for elderly hip-fracture patients with CKD unless clinically contraindicated; dosing and agent selection were individualized by the treating physicians with consideration of renal function. Preoperative age, WBC, and D-dimer were not used to decide whether to initiate or withhold prophylaxis and were analyzed only as potential risk markers.

This study involved 180 participants, including 62 men and 118 women. We divided them into two groups: the DVT group, consisting of patients diagnosed with deep vein thrombosis, and the non-DVT group, comprising individuals without DVT, to streamline our analysis. Throughout their stay in the hospital, all patients were provided with both mechanical and chemical preventive measures against DVT. Chemoprophylaxis was primarily achieved through the administration of low-molecular-weight heparin sodium (LMWH) injections. Specifically, LMWH was administered routinely at a dose of 4250 IU once daily, starting from the time of hospitalization until 12 h before surgery, and then resumed 12 h post-surgery until discharge. Patients diagnosed with DVT received an intensified regimen of LMWH sodium, at a dosage of 4250 IU every 12 h. Mechanical prophylaxis was implemented using a pneumatic compression device applied intermittently during their hospital stay. Upon admission, color Doppler ultrasounds were conducted on both lower limbs of all patients to screen for DVT, with the diagnosis confirmed by at least two ultrasound specialists. All DVT cases recorded in this study were diagnosed preoperatively, prior to surgical intervention. Patients who developed postoperative DVT were not included in this analysis. Additionally, DVT location was classified as proximal (involving the popliteal, femoral, or iliac veins) or distal (involving the calf veins). Notably, in this study, none of the patients required an inferior vena cava filter or experienced PE.

In this study, we conducted a thorough review of patient demographics, comorbidities, and initial laboratory test results upon admission (all samples were collected during the first blood draw upon hospital admission, before any surgical intervention or anticoagulant administration, to ensure that baseline thrombotic status was assessed). The demographic data included age, gender, body mass index (BMI), the time elapsed from injury to hospital admission, and whether a blood transfusion was performed. Additionally, we explored a range of comorbidities, such as anemia, hypoproteinemia, hypertension, diabetes, dysuria, heart failure, respiratory failure, and delirium, to gain a comprehensive understanding of each patient’s health background. Our analysis also extended to a detailed examination of various laboratory indicators at the time of admission. These included hemoglobin (HGB), hematocrit (HCT), lymphocyte count (LYM), mean corpuscular hemoglobin (MCH), mean corpuscular hemoglobin concentration (MCHC), mean corpuscular volume (MCV), mean platelet volume (MPV), neutrophil count (NEU), platelet count (PLT), red blood cell count (RBC), and white blood cell count (WBC). Coagulation-related measures, such as activated partial thromboplastin time (APTT), D-Dimer, fibrinogen (FIB), prothrombin time (PT), international normalized ratio (INR), and thrombin time (TT), were also included. Moreover, we assessed a variety of biochemical markers, encompassing albumin (ALB), aspartate aminotransferase (AST), alanine transaminase (ALT), calcium (Ca), creatine kinase (CK), creatine kinase-MB (CKMB), creatinine (CREA), total bilirubin (tbil), direct bilirubin (DBIL), indirect bilirubin (IBIL), glucose (GLU), lactic dehydrogenase (LDH), triglyceride (TG), total protein (TP), urea (UREA), and uric acid (UA).

### Statistics

In this study, we employed SPSS software (version 25.0, SPSS Inc., New York, USA) and considered a significance level of *p* < 0.05. For continuous variables, we first conducted the Shapiro–Wilk test to assess normality. If the data met the normality assumption, we reported these variables as mean ± SD (standard deviation) and used the *t* test for group comparisons. Alternatively, if the normality assumption was not met, we applied the Mann–Whitney *U* test for statistical analysis between the two groups.

To examine differences between groups for categorical variables, we utilized the Chi-square and Fisher’s exact tests, reporting the results as counts and percentages. Furthermore, in our quest to identify the most robust predictors of DVT, we conducted binary logistic regression analysis to identify independent predictors of DVT in patients who were diagnosed with both hip fractures and CKD.

To determine optimal cut-off values for continuous variables like age, we routinely conducted ROC (receiver-operating characteristic) analysis, seeking the point where the Youden index (sensitivity + specificity—1) reached its maximum. These cut-off values were used to classify individuals into low- and high-risk categories. The diagnostic performance was assessed using the area under the ROC curve (AUC), which ranged from 0 to 100%, with a larger AUC indicating better diagnostic accuracy.

To assess potential collinearity between age and D-dimer levels, we conducted Pearson correlation analysis and variance inflation factor (VIF) calculation. A VIF threshold > 5 was considered indicative of high collinearity.

## Result

Between November 2015 and January 2023, our study enrolled a total of 180 patients who had both hip fractures and CKD, all of whom met the specified inclusion criteria. Among these participants, there were 118 females, constituting 65.6% of the cohort, while the remaining 62 patients, accounting for 34.4%, were males. The median age for this patients was 76 years. Regarding the incidence of DVT, it was found to be 34.4%, with 62 patients diagnosed with DVT, while the remaining 118 did not exhibit this condition. No cases of postoperative DVT were included in this study. Regarding DVT location, 40 patients (64.5%) had distal DVT, while 22 patients (35.5%) had proximal DVT.

Since D-dimer levels tend to increase with age, we examined potential collinearity between age and D-dimer. However, our analysis showed that there was no significant correlation (*r* = 0.001,* p* = 0.988), and VIF values remained low (1.000), confirming that collinearity did not affect our logistic regression model. These findings suggest that age and D-dimer independently contribute to DVT risk in elderly patients.

As shown in Table 1, a noteworthy difference emerged between the DVT group and the non-DVT group with regard to age (*p* = 0.031). It is evident from the table that the DVT group exhibited a higher age compared to the non-DVT group. However, when considering gender, BMI, the time elapsed from injury to hospital admission, and blood transfusions, as well as comorbidities such as anemia, hypoproteinemia, hypertension, diabetes, dysuria, heart failure, respiratory failure, and delirium, there were no significant differences observed between these two groups (all *p *> 0.05).

**Table 1:** Demographics and comorbiditie’s data of patients with and without DVT

Demographics	DVT group (*n* = 62)	Non-DVT group (*n* = 118)	*p*
Age, years	79.5 (74.0 ~ 84.0)	77.0 (69.0 ~ 82.3)	0.031*
Gender, *n* (%)			
Male	19 (26.4%)	43 (39.8%)	0.063
Female	53 (73.6%)	65 (60.2%)	
BMI, kg/m2	23.5 (21.9 ~ 24.9)	23.9 (22.7 ~ 24.8)	0.075
Time from injury to admission, hours			
< 1d	36 (58.1%)	76 (64.4%)	0.701
1–3d	14 (22.6%)	22 (18.6%)	
≥ 3d	12 (19.4%)	30 (16.9%)	
Transfusion, *n* (%)			
Yes	32 (51.6%)	51 (43.2%)	0.283
No	30 (48.4%)	67 (56.8%)	
Comorbidities			
Anemia, *n* (%)			
Yes	30 (48.4%)	73 (61.9%)	0.082
No	32 (51.6%)	45 (38.1%)	
Hypoproteinemia, *n* (%)			
Yes	40 (64.5%)	74 (62.7%)	0.811
No	22 (35.5%)	44 (37.3%)	
Hypertension, *n* (%)			
Yes	46 (74.2%)	77 (65.3%)	0.221
No	16 (25.8%)	41 (34.7%)	
Diabetes, *n* (%)			
Yes	26 (41.9%)	46 (39.0%)	0.701
No	36 (58.1%)	72 (61.0%)	
Dysuresia, *n* (%)			
Yes	13 (21.0%)	17 (14.4%)	0.262
No	49 (79.0%)	101 (85.6%)	
Heart failure, *n* (%)			
Yes	8 (12.9%)	14 (11.9%)	0.840
No	54 (87.1%)	104 (88.1%)	
Respiratory failure, *n* (%)			
Yes	10 (16.1%)	10 (8.5%)	0.120
No	52 (83.9%)	108 (91.5%)	
Delirium, *n* (%)			
Yes	2 (3.2%)	3 (2.5%)	0.791
No	60 (96.8%)	115 (97.5%)	

*BMI* body mass index; values are presented as the number (%) or the median (interquartile range). *p < 0.05, statistical significance

Table 2 presents a summary of the laboratory findings for both the DVT group and the non-DVT group. In the DVT group, notably, the levels of WBC (*p* = 0.005), thrombin time (TT) (*p* = 0.030), and D-dimer (*p* < 0.001) were significantly elevated compared to the non-DVT group. Nevertheless, no statistically significant differences were observed between these two groups in terms of other laboratory results (all *p* > 0.05).

**Table 2:** Laboratory results of patients with and without DVT

Laboratory results	DVT group (*n* = 62)	Non-DVT group (*n* = 118)	*p*
HCT	31.75 (28.26 ~ 34.63)	31.75 (28.08 ~ 36.39)	0.532
HGB	105.22 (95.13 ~ 113.73)	105.22 (91.75 ~ 119.25)	0.416
LYM	1.19 (1.16 ~ 1.23)	1.18 (1.15 ~ 1.24)	0.755
MCH	30.55 (29.78 ~ 31.82)	30.54 (29.22 ~ 31.44)	0.658
MCHC	331.76 (328.88 ~ 336.25)	331.76 (325.15 ~ 339.60)	0.970
MCV	92.10 (89.11 ~ 96.04)	92.10 (88.78 ~ 95.03)	0.970
MPV	9.81 (9.38 ~ 10.30)	9.81 (9.08 ~ 10.73)	0.654
PLT	214.68 (185.83 ~ 249.35)	214.68 (153.93 ~ 244.50)	0.233
RBC	3.46 (3.12 ~ 3.75)	3.46 (3.08 ~ 3.93)	0.744
WBC	9.87 (8.17 ~ 12.53)	9.01 (7.01 ~ 9.96)	0.005*
APTT	29.63 (27.60 ~ 30.50)	29.10 (26.50 ~ 29.66)	0.165
D-dimer	3.33 (3.27 ~ 4.96)	3.30 (1.67 ~ 3.30)	< 0.001*
FIB	4.07 (3.57 ~ 4.80)	4.06 (3.31 ~ 4.58)	0.151
INR	1.09 (1.05 ~ 1.11)	1.09 (1.02 ~ 1.12)	0.820
PT	12.41 (12.00 ~ 12.60)	12.50 (11.50 ~ 12.68)	0.852
TT	17.30 (16.48 ~ 17.33)	17.29 (16.15 ~ 17.50)	0.030*
ALB	34.75 (31.47 ~ 37.33)	34.70 (33.21 ~ 37.40)	0.860
ALT	15.25 (9.87 ~ 24.57)	21.90 (10.78 ~ 24.55)	0.151
AST	20.60 (14.00 ~ 32.77)	23.10 (15.46 ~ 32.80)	0.064
Ca	2.15 (2.09 ~ 2.15)	2.15 (2.15 ~ 2.18)	0.549
CK	139.21 (81.16 ~ 249.54)	248.91 (86.38 ~ 248.91)	0.532
CKMB	18.15 (9.24 ~ 19.24)	19.23 (12.80 ~ 19.23)	0.633
CREA	150.00 (86.86 ~ 225.40)	224.68 (92.11 ~ 225.15)	0.431
TBIL	15.43 (11.03 ~ 19.28)	15.65 (11.15 ~ 16.28)	0.884
DBIL	5.60 (3.45 ~ 6.54)	5.90 (3.56 ~ 5.90)	0.826
IBIL	9.36 (5.65 ~ 11.80)	9.38 (6.40 ~ 10.05)	0.942
GLU	8.28 (5.54 ~ 8.65)	8.65 (6.46 ~ 8.65)	0.363
LDH	365.05 (221.60 ~ 376.86)	371.43 (216.53 ~ 371.43)	0.093
TG	1.24 (0.97 ~ 1.24)	1.24 (1.10 ~ 1.24)	0.891
TP	62.15 (58.90 ~ 65.55)	62.07 (59.15 ~ 66.05)	0.805
UA	370.69 (295.30 ~ 469.70)	370.73 (312.00 ~ 386.55)	0.962
Urea	12.75 (7.74 ~ 14.79)	13.00 (7.89 ~ 14.91)	0.316

*HCT* hematocrit, *HGB* hemoglobin, *LYM* lymphocyte, *MCH* mean corpuscular hemoglobin, *MCHC* mean corpuscular hemoglobin concentration, *MCV* mean corpuscular volume, *MPV *mean platelet volume, *PLT* platelet, *RBC *red blood cell, *WBC* white blood cell, *APTT *activated partial thromboplastin time, *FIB *fibrinogen, *INR *international normalized ratio, *PT *prothrombin time, *TT *thrombin time, *ALB* albumin, *AST* aspartate aminotransferase, *ALT* alanine transaminase, *Ca* calcium, *CK* creatine kinase, *CKMB *creatine kinase-MB, *CREA *creatinine, *TBIL *total bilirubin, *IBIL *indirect bilirubin, *DBIL *direct bilirubin, *GLU* glucose, *LDH* lactic dehydrogenase, *TG* triglyceride, *TP* total protein, *UREA *ureophil, *UA* uric acid, values are presented as the number (%) or the median (interquartile range). *p < 0.05, statistical significance

As per the logistic regression analysis findings, there is a significant association between DVT and certain factors among patients diagnosed with hip fractures and CKD, as illustrated in Table 3. Specifically, older age [*p* = 0.009, OR = 1.049, 95% CI (1.012, 1.087)], elevated WBC levels [*p* = 0.001, OR = 1.131, 95% CI (1.054, 1.214)], and higher D-dimer levels [*p* = 0.010, OR = 1.190, 95% CI (1.043–1.358)] were identified as closely linked to the occurrence of DVT in these patients.

**Table 3:** Binary logistic regression analysis of variables associated with DVT

Characteristics	OR	95%CI	*p*
Age	1.049	1.012–1.087	0.009*
WBC	1.131	1.054–1.214	0.001*
D-dimer	1.190	1.043–1.358	0.010*

*WBC*   white blood cell. *p < 0.05, statistical significance

**Fig. 2:**
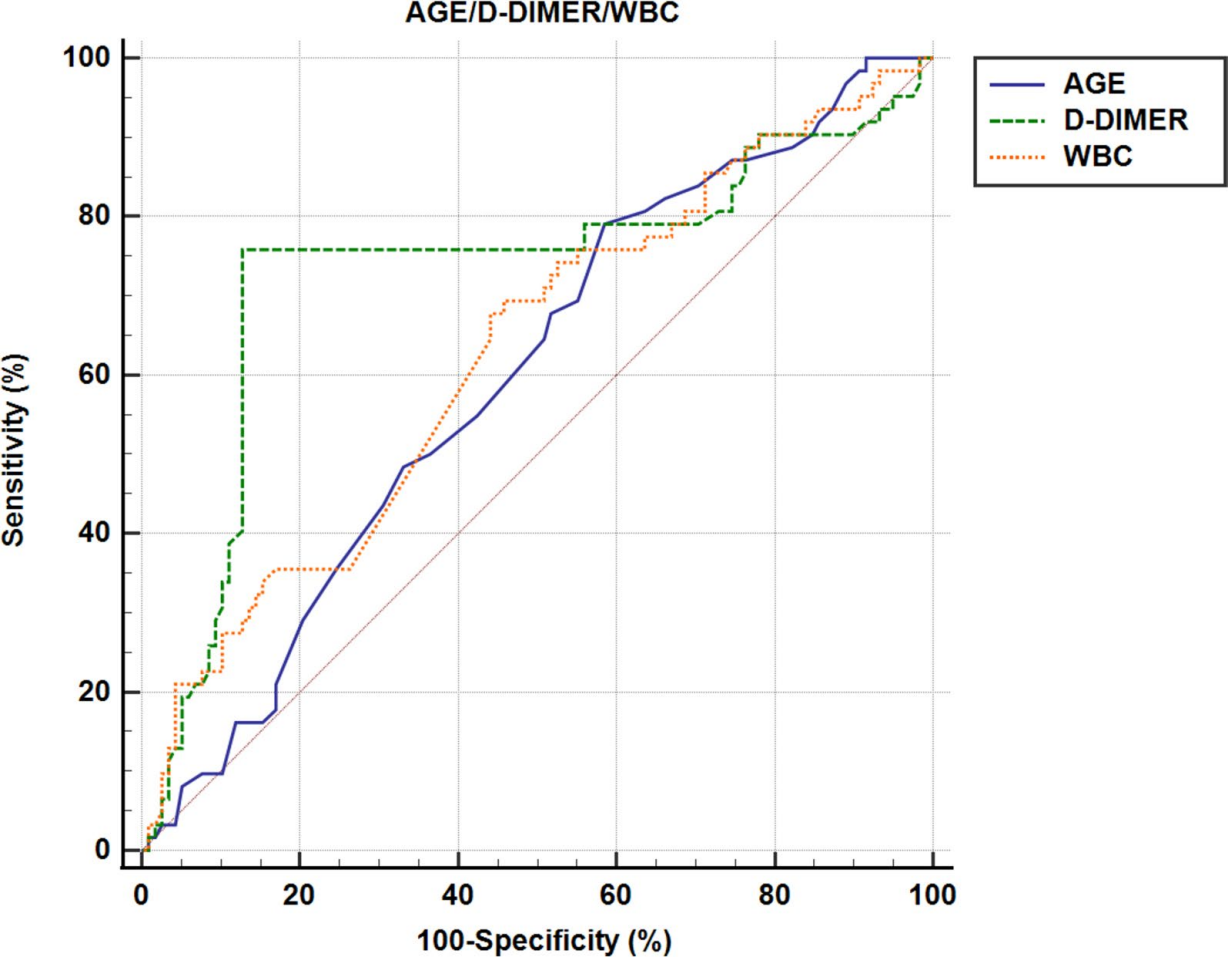
ROC curve for AGE/D-DIMER/WBC

**Fig. 3:**
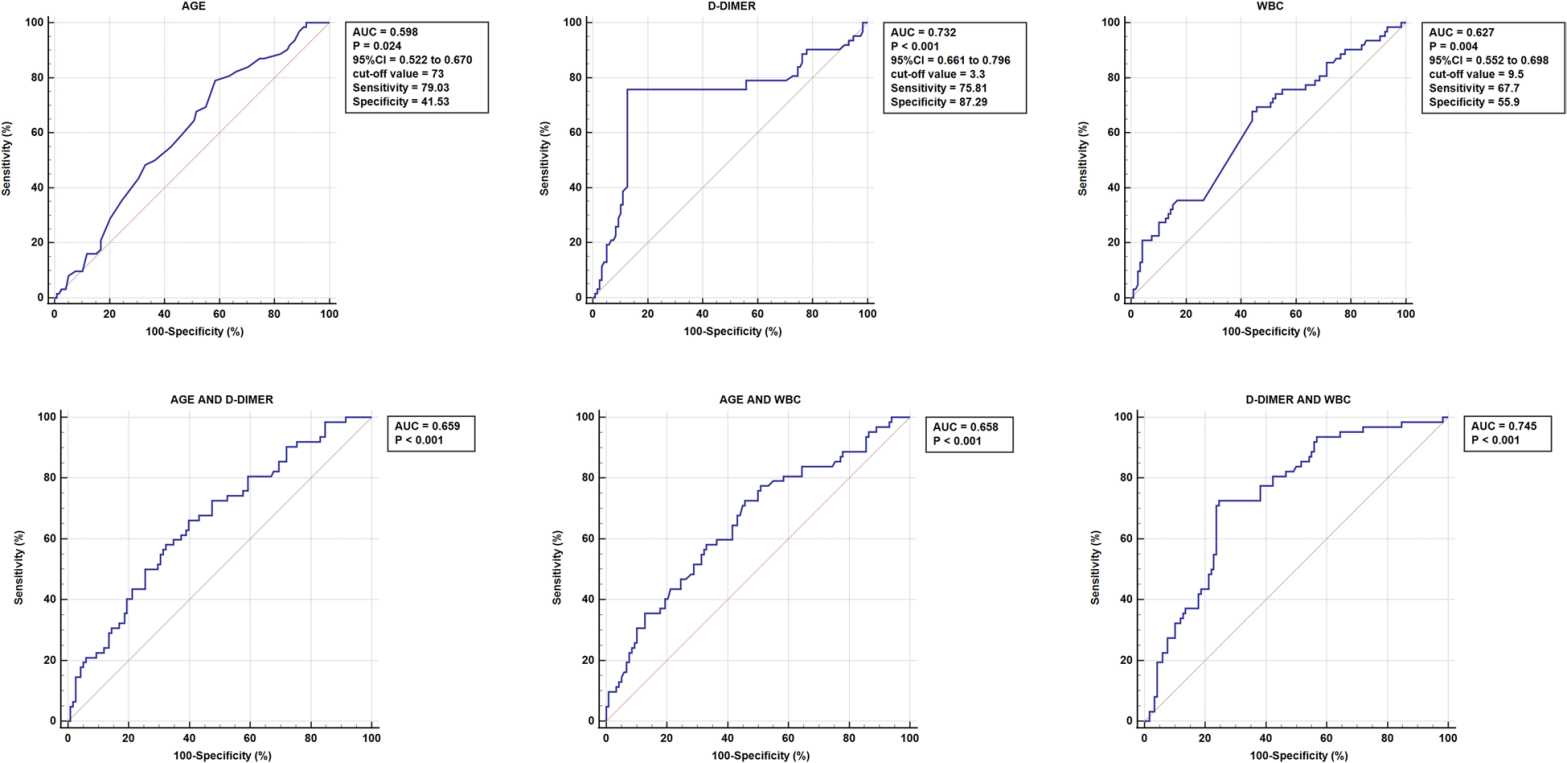
ROC curve for seven different indices

**Fig. 4:**
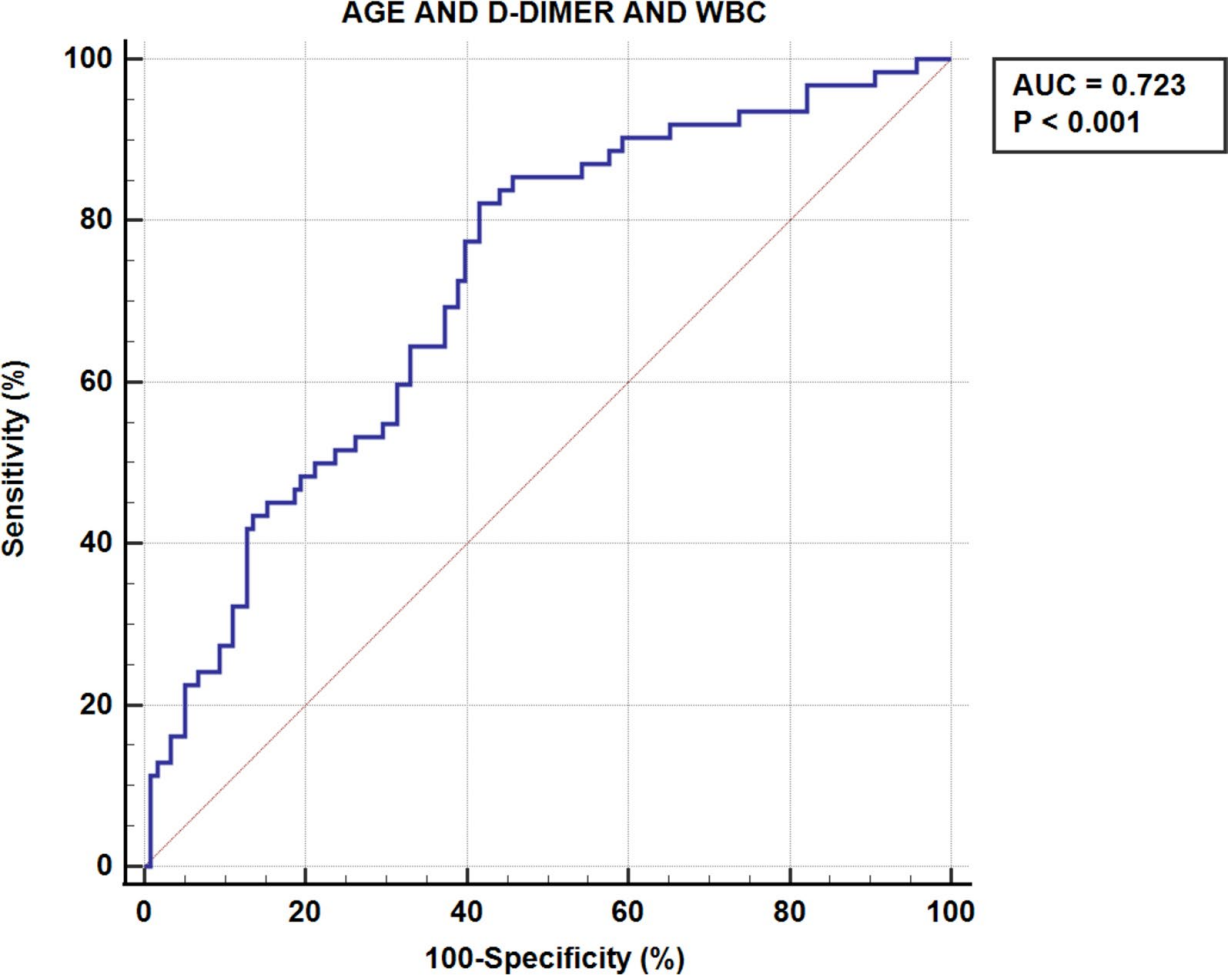
ROC curve for AGE+D-DIMER+WBC

The findings from the ROC curve analysis, as illustrated in Fig. 2, underscored age, WBC, and D-dimer as robust and independent predictors of DVT. The optimal cut-off points for these variables were determined to be 73 years for age, 9.5 × 10^9/μL for WBC, and 3.3 ng/mL for D-dimer. Figure 3 provides a comprehensive visualization of the AUC for seven different indices, offering valuable insights into their predictive abilities. Age alone exhibited statistical significance (*p* = 0.024) with an AUC area of 0.598 (95% CI 0.522–0.670), while WBC alone also demonstrated significance (*p* = 0.004) with an AUC area of 0.627 (95% CI 0.552–0.698). Notably, D-dimer alone displayed the highest predictive power (*p* < 0.001) with an impressive AUC area of 0.732 (95% CI 0.661–0.796). Moreover, combining age with D-dimer (*p* < 0.001, AUC area = 0.659), age with WBC (*p* < 0.001, AUC area = 0.658), and WBC with D-dimer (*p* < 0.001, AUC area = 0.745) significantly enhanced the accuracy of DVT prediction. The most comprehensive model, incorporating all three factors (age, WBC, and D-dimer), also yielded a highly significant AUC area of 0.723 (*p* < 0.001). These results emphasize the importance of considering these variables collectively for a more robust risk assessment of DVT Fig. 4.

## Discussion

Hip fractures significantly impact the health and quality of life in the elderly. The prevalence of CKD is rising among this demographic. As kidney function declines, there is an observed increase in fracture rates in older individuals, especially following falls [2]. ROD, a major public health concern in aging populations, further complicates the management of hip fractures in CKD patients. This condition not only intensifies the severity of fractures but also challenges post-fracture recovery, significantly affecting overall patient health [3]. Elderly patients with hip fractures and CKD often experience severe pain and reduced mobility, leading to prolonged recovery periods. The combination of these conditions, particularly decreased mobility and the systemic effects of CKD, not only impairs overall health but also markedly elevates the risk of DVT as an additional complication. DVT is not only a frequent complication in older individuals with hip fractures, but it is also one of the most common complications in hospitalized patients, leading to disability and even death in severe cases [11]. Despite improvements in medical diagnostics reducing DVT rates, it remains a concern. Recent studies show that 19.5–32% of hip-fracture patients have DVT before surgery [12]. Our study found a 34.4% preoperative DVT rate in elderly hip fracture patients, a significant figure. Key reasons include the older age group’s inherent risk, the hip’s vulnerability to DVT, and the added risk from coexisting CKD, which may increase DVT likelihood [13]. Although there are existing studies exploring the risk factors of DVT preoperatively in elderly patients with hip fractures, and some have even identified CKD as a risk factor for DVT in these patients, research specifically targeting the population of elderly hip-fracture patients who already have CKD remains limited [7, 13]. There is still a research gap in understanding the risk factors of DVT in this particular demographic.

The prevalence of CKD is steadily increasing in the general population, and it frequently coexists as a comorbidity in patients who suffer from hip fractures [14]. In the previous studies, it was established that patients with CKD faced a heightened risk of preoperative DVT occurrence compared to those with normal kidney function [15, 16]. After total joint arthroplasty, Li et al. observed that CKD can elevate the risk of both total and symptomatic DVT. Specifically, they reported a relative risk of 2.68 for DVT in patients with stage 3/4 CKD. Besides, in their report, Lutz et al. noted that the risk of thromboembolic disease was 2.5 times higher in individuals with mild renal dysfunction and 5.5 times higher in those with severe renal dysfunction when compared to patients with normal kidney function. In patients with CKD, the hemostatic system undergoes various alterations. This includes changes in platelet function and levels of coagulation factors, leading to a hypercoagulable state and thereby increasing the risk of thrombus formation. Moreover, CKD often coexists with other conditions, such as hypertension and diabetes, which are themselves risk factors for thrombosis [17]. Despite the established links between CKD and an increased risk of DVT in such patients, there remains a significant gap in the literature specifically addressing the cumulative risk factors for DVT in elderly patients with hip fractures and CKD. This study aims to fill this critical gap by systematically evaluating the various preoperative risk factors that contribute to the incidence of lower limb DVT in this specific patient population. Understanding these unique risk factors is essential for developing targeted preventive strategies and improving patient outcomes in this vulnerable group.

In our current study, we observed that out of 180 participants, 62 individuals, or 34.4%, experienced DVT. Our logistic regression analysis indicated that older age, higher WBC, and elevated D-dimer levels were associated with an increased risk of DVT. Specifically, the cut-off values for predicting DVT, as determined by ROC curve analysis, were found to be 73 years for age, 9.5 × 10^9/μL for WBC count, and 3.3 ng/mL for D-dimer levels.

D-dimer is a blood test used to assess the risk of DVT. It is a product of fibrin breakdown in blood clots. Thrombin cleaves fibrinogen into fibrin monomers, which then form fibrin polymers. Factor XIIIa cross-links D domains, and plasmin breaks down fibrin, releasing D-dimer [18]. In DVT diagnosis, D-dimer level measurement is key. Normal D-dimer often excludes DVT, but high levels are not specific and may be caused by other factors like infections or trauma. Therefore, high D-dimer necessitates further evaluation with clinical symptoms and additional diagnostic methods [19]. This research revealed that in patients who suffer from both elderly hip fractures and CKD, those diagnosed with DVT exhibit significantly higher levels of D-dimer compared to those not afflicted with DVT. The establishment of a threshold level at 3.3 ng/mL for D-dimer is a key finding of this study. Exceeding this threshold suggests a heightened risk of DVT in such patients, thereby necessitating closer monitoring and potentially more aggressive preventative strategies for those whose D-dimer levels surpass this critical value. A number of previous studies have established a significant correlation between elevated D-dimer levels and the occurrence of preoperative DVT in patients with hip fractures [20–22]. Furthermore, studies have also revealed a relationship between heightened D-dimer levels and an increased risk of DVT in individuals afflicted with CKD, emphasizing the broader relevance of D-dimer as an indicator in diverse health conditions [23]. The findings from these studies are consistent with the results of our own research. This might be due to elevated levels of D-dimer, a fibrin degradation product indicative of increased fibrinolytic activity typically spurred by clot formation, serving as a predictive biomarker for DVT and reflecting thrombotic processes within the body. Consequently, in clinical practice, heightened vigilance for the potential occurrence of DVT is imperative in these patients exhibiting raised D-dimer levels.

Advanced age is commonly considered a significant risk factor for the occurrence of DVT [24, 25]; however, our study provides additional insight by identifying a specific cut-off value (≥ 73 years) for increased DVT risk in elderly patients with hip fractures and CKD. This age threshold may assist in refining risk prediction models and guiding individualized thromboprophylaxis strategies in this vulnerable population. Barco et al. conducted a comprehensive study involving 24,911 patients who were objectively diagnosed with a first episode of lower limb DVT, demonstrating that the risk of DVT depends on a combination of factors, including age, gender, and other VTE risk factors [24]. Another study by Stein et al. emphasized the impact of an aging population on the rate ratios for the diagnosis of DVT. This research indicated that over a span of 21 years, the rate of DVT diagnosis increased exponentially [26]. These findings underscore the significant role that age plays in the incidence of DVT, which is consistent with our research. In addition to this, our study also found that when the age of the patients included in this study exceeded 73 years, the risk of developing DVT significantly increased. As individuals age, a combination of factors comes into play, including the stiffening of blood vessels, potentially leading to a reduction in blood flow, especially in the veins of the lower limbs. This diminished flow rate can result in prolonged blood stagnation within the veins, consequently increasing the likelihood of clot formation. Furthermore, the aging process can lead to functional impairment in vascular endothelial cells and alterations in the mechanisms governing blood coagulation, further contributing to the heightened risk of clot formation. Additionally, age-related changes in blood components, such as an elevation in fibrinogen levels, can enhance susceptibility to clot formation, thereby elevating the overall risk of developing DVT. Therefore, clinical management should emphasize age-specific risk assessment and tailored preventive measures to address this elevated risk effectively.

Our study found that among the patients included in this research, the DVT group exhibited higher WBC levels compared to the non-DVT group, which is consistent with the findings of many other studies. In Hou et al.’s brief review, it is demonstrated that the contribution of biomarkers, including WBC, to the diagnosis and guidance of therapy for DVT is significant [27]. Additionally, Passamonti et al. conducted an evaluation of the impact of the increase in WBC over time on the risk of thrombosis. Their study revealed that patients who experienced an increase in WBC within the first 2 years after diagnosis had a higher risk of thrombosis compared to patients with stable WBC [28]. Furthermore, in Chen et al.’s prospective cross-sectional study, higher WBC and LDH levels may be associated with thrombosis, as they were significantly elevated in the VTE group compared to the non-VTE group [29]. Stimulation by various agents can activate blood leukocytes, leading to the development of potent procoagulant activity that can trigger the extrinsic pathway of blood clot formation. Furthermore, it has been established that early events in the initiation of DVT involve leukocyte adhesion and transmigration [30, 31]. It is important to note that an elevated WBC level is a non-specific indicator and cannot be used for the direct diagnosis of DVT. Further research is needed to confirm the impact of WBC on the formation of DVT.

It is well recognized that elevated WBC and D-dimer levels may be influenced by multiple factors, including infections, inflammation, and recent trauma. However, despite their non-specific nature, our findings suggest that in elderly patients with hip fractures and CKD, these markers still provide meaningful predictive value when considered collectively. Previous studies have also indicated that increased WBC contributes to a hypercoagulable state by promoting endothelial dysfunction and activating the extrinsic coagulation pathway [27–29]. Similarly, D-dimer, as a fibrin degradation product, is widely used in DVT screening, although its specificity remains limited [18, 19]. Therefore, while these markers should not be used in isolation to diagnose DVT, our study highlights their utility in combination with other clinical parameters for risk stratification.

Our findings identify age, D-dimer, and WBC as non-specific but informative markers of preoperative DVT risk. Given their susceptibility to multiple comorbid influences, these variables should not be used to withhold pharmacologic thromboprophylaxis in elderly hip-fracture patients with CKD. Instead, they may prioritize surveillance (e.g., closer clinical observation or repeat duplex ultrasonography) and considerations for tailored or extended prophylaxis, while pharmacologic prophylaxis remains generally warranted unless contraindicated.

While this study presents several innovative findings, it is essential to acknowledge certain limitations. First, it was conducted at a single center, indicating the need for a multicenter clinical study with a larger sample size. Second, given its retrospective nature, this study had limited access to certain potential variables associated with DVT risks, such as prior surgical history. Additionally, like any multivariate analysis, we could not account for all confounding factors, leaving residual confounding as a potential issue. Third, WBC and D-dimer are non-specific markers, and their elevated levels do not necessarily indicate DVT. Future prospective studies with serial biomarker measurements and additional inflammatory markers may help refine their role in DVT prediction. Fourth, our study involved multiple variables to assess DVT risk, using binary logistic regression. However, we did not specifically adjust for the multiplicity of over 40 covariates, which could raise the chance of finding significant results by chance. Future research should consider methods to control for this issue to ensure more reliable conclusions. Fifth, although all patients in this cohort received standardized pharmacologic and mechanical thromboprophylaxis, potential variations in dose adjustments, timing, or adherence could not be fully captured in this retrospective design and may have influenced the observed risk of DVT. Finally, CKD stage could not be assessed, because stage information was not routinely documented in the EMR, and albuminuria/standardized eGFR data were incomplete in this retrospective cohort. As a result, we were unable to determine stage-specific DVT risk within CKD, which may reduce the granularity of risk estimates.

In conclusion, our study identified age, D-dimer, and WBC levels as independent predictors of DVT through logistic regression analysis. We determined the optimal cut-off values for these variables to be 73 years for age, 9.5 × 10^9/μL for WBC, and 3.3 ng/mL for D-dimer. These findings offer a personalized assessment of DVT risk in elderly patients with concurrent hip fractures and CKD, enabling early targeted interventions.

## Data Availability

No datasets were generated or analysed during the current study.

## Abbreviations

DVT: Deep venous thrombosis
CKD: Chronic kidney disease
ROD: Renal osteodystrophy
VTE: Venous thromboembolism
PE: Pulmonary embolism
ROC: Receiver-operating characteristic curve
AUC: Area under the curve
BMI: Body mass index
HCT: Hematocrit
HGB: Hemoglobin
LYM: Lymphocyte
MCH: Mean corpuscular hemoglobin
MCHC: Mean corpuscular hemoglobin concentration
MCV: Mean corpuscular volume
MPV: Mean platelet volume
PLT: Platelet
RBC: Red blood cell
WBC: White blood cell
APTT: Activated partial thromboplastin time
FIB: Fibrinogen
INR: International normalized ratio
PT: Prothrombin time
TT: Thrombin time
ALB: Albumin
AST: Aspartate aminotransferase
ALT: Alanine transaminase
Ca: Calcium
CK: Creatine kinase
CKMB: Creatine kinase-MB
CREA: Creatinine
TBIL: Total bilirubin
IBIL: Indirect bilirubin
DBIL: Direct bilirubin
GLU: Glucose
LDH: Lactic dehydrogenase
TG: Triglyceride
TP: Total protein
UREA: Ureophil
UA: Uric acid
