# Correction: Evaluating preoperative risk factors for deep vein thrombosis in elderly patients with hip fractures and chronic kidney disease: a retrospective study

**DOI:** 10.1186/s40001-026-03866-3

**Published:** 2026-01-22

**Authors:** Ruili Jia, Xiaoqian Men, Fang Ran, Xiaodong Li, Yubin Long

**Affiliations:** 1 Department of Nephrology, Baoding First Central Hospital of Hebei Medical University, Baoding, China; 2 Department of Ultrasound, The Third Hospital of Hebei Medical University, Shijiazhuang, China; 3 Department of Orthopedics, Baoding First Central Hospital, Baoding, China

**Correction: European Journal of Medical Research (2025) 30:1153** 10.1186/s40001-025-03409-2

In this article the affiliation details for Yubin Long was incorrectly given as ‘Department of Orthopedic Surgery, The Third Hospital of Hebei Medical University, Shijiazhuang, China’but should have been

‘Department of Orthopedics, Baoding First Central Hospital, Baoding, China’.

The original article has been corrected.

